# Preoperative risk factors of lymph node metastasis in clinical N0 lung adenocarcinoma of 3 cm or less in diameter

**DOI:** 10.1186/s12893-022-01605-z

**Published:** 2022-04-29

**Authors:** Cheng Fang, Yangwei Xiang, Weili Han

**Affiliations:** Department of Lung Transplantation, The First Affiliated Hospital, Zhejiang University School of Medicine, No.79 Qingchun Road, Hangzhou, 310003 Zhejiang Province China

**Keywords:** Lung adenocarcinoma, Risk factor, Lymph node metastasis, Lymph node dissection

## Abstract

**Background:**

Lung adenocarcinoma is the most common subtype of non-small cell lung cancer. The surgical strategy of lymph node dissection is controversial because many more patients are diagnosed at an early stage in clinical practice.

**Methods:**

We retrospectively reviewed 622 clinical N0 lung adenocarcinoma patients with 3 cm or less in tumor size who underwent lobectomy or segmentectomy combined with lymph node dissection in our hospital from January 2017 to December 2019. We performed univariate and multivariate analyses to identify preoperative risk factors of lymph node metastasis.

**Results:**

Lymph node metastasis was found in 60 out of 622 patients. On univariate analysis, lymph node metastasis was linked to smoking history, preoperative CEA level, tumor size, tumor location (peripheral or central), consolidation/tumor ratio, pleural invasion, and pathologic type. However, only the preoperative CEA level, tumor size, and consolidation/tumor ratio were independent risk factors in multivariate analysis. The ROC curve showed that the cutoff value of tumor size was 1.7 cm. There was no lymph node metastasis in patients without risk factors.

**Conclusions:**

The preoperative CEA level, tumor size, and consolidation/tumor ratio were independent risk factors of lymph node metastasis in clinical N0 lung adenocarcinoma with tumor size ≤ 3 cm. The lymph node metastasis rate was extremely low in clinical N0 lung adenocarcinoma patients without risk factors and lymph node dissection should be avoided in these patients to reduce surgical trauma.

## Background

Lung cancer is a common disease with high mortality rate all over the world. According to statistics, lung cancer accounts for 11.4% of the worldwide new cases of cancer and 18% of cancer deaths [1]. Non-small cell lung cancer is the most common type, in which squamous cell carcinoma and adenocarcinoma account for the vast majority. Nowadays, lung adenocarcinoma has become the most common type of non-small cell lung cancer surpassing squamous cell carcinoma.

Lymph node metastasis is an important site for metastasis of lung cancer and directly affects the patient survival. The accurate N stage is important in guiding postoperative treatment and predicting postoperative survival. The standard surgical treatment for lung cancer used to be lobectomy and systematic lymph node dissection. However, with the wide application of high resolution computer tomography (HRCT), many early stage lung cancers have been diagnosed, with ground-grass lesion as the main feature. According to the literature, lung cancer with pure ground grass opacity (GGO) does not present with lymph node metastasis, and the incidence of lymph node metastasis was low in GGO-dominant lung cancer [2, 3]. Systematic lymph node dissection is actually unnecessary and only increases surgical trauma in patients with early stage lung cancer. However, it is hard to know the accurate stage of lymph node by conventional examinations before operation. Liberman et al. reported a sensitivity and specificity of 12.8% and 99.6%, respectively for the diagnosis of lymph node metastasis on plain CT, while the sensitivity and specificity was 22.1% and 90.7%, respectively on contrast CT. The sensitivity and specificity was 39.7% and 80.3%, respectively using positron emission tomography/computed tomography (PET/CT) in the diagnosis of lymph node metastasis [4].

From literature several risk factors are related to lymph node metastasis such as age [5], tumor size [6, 7], carcinoembryonic antigen (CEA) level [8], consolidation/tumor ratio (CTR) [9], tumor location [10], poor differentiation [11], pleural invasion [12], lymphatic vascular invasion [13], micropapillary component [14], and tumor spread through air spaces [15]. However, characteristics such as the micropapillary component, lymphatic vascular invasion, degree of differentiation, and tumor spread through air spaces were often unconfirmed until one week after surgery in paraffin-embedded pathology specimens. Surgeons can only obtain the results of preoperative examinations, intraoperative exploration, and fast frozen pathology during surgery before lymph node dissection. Fast frozen pathology during surgery in our hospital can only reveal the type of lung cancer (e.g. squamous cell carcinoma or adenocarcinoma) and also the invasive degree of the tumor (adenocarcinoma in situ, micro invasive and invasive adenocarcinoma). Lepidic-predominant adenocarcinoma can also be diagnosed by fast frozen pathology during surgery in our hospital.

Presently, the necessity of lymph node dissection of clinical early stage lung cancer remains controversial and lacks a consensus in clinical practice [16, 17]. We analyzed the preoperative characteristics of lung adenocarcinoma combined with tumor invasive degree which can be revealed by fast frozen pathology during surgery, and we tried to find the risk factors of lymph node metastasis of lung adenocarcinoma and provide reference data for clinical practice.

## Methods

### Patient selection

We retrospectively reviewed the clinical data of patients operated for lung cancer in our hospital from January 2017 to December 2019. We included (1) All patients should receive conventional preoperative examinations to exclude distant metastasis. High resolution CT should be performed one month before surgery; (2) The pathology was confirmed as lung adenocarcinoma; (3) The clinical N stage before operation should be N0 and the maximum tumor diameter on CT image should be 3 cm or less; and (4) Lobectomy or segmentectomy combined with lymph node dissection was performed. The number of patients received segmentectomy was increasing in recent years in our center. At present, surgeons in our hospital tend to perform sublobar resection instead of lobectomy for clinical early stage patients with GGO-dominant lension and tumor size ≤ 2 cm. The surgical margin should not be less than 2 cm or the maximum diameter of the tumor. Patients with poor pulmonary function who cannot bear lobectomy also receive sublobar resection. Lymph node dissection should include at least three N1 stations (always contain stations 10, 11 and 12) and at least three N2 stations (always contain subcarinal station) in accordance with the criteria raised by the European Society of Thoracic Surgeons [18]. Lobe specific lymph node stations should also be included. We excluded: (1) patients who had received neoadjuvant therapy before surgery (chemotherapy, immunotherapy or targeted therapy); (2) patients with multiple malignant lesions; (3) carcinoma in situ; (4) parietal pleura or pericardium invaded; (5) more than one lobe invaded; (6) history of any other malignant tumors; and (7) lung adenocarcinoma associated with cystic airspaces which was hard to measure the exact GGO or solid component. We included 622 patients in the study.

### Data collection

Patient information was collected from the electronic database in our hospital. We collected the clinical data such as gender, age, smoking history, preoperative CEA level, surgery type, tumor location, pathologic characteristic, tumor size, consolidation/tumor ratio, and lymph node metastasis for analysis. Consolidation/tumor ratio was calculated as the proportion of the maximum consolidation diameter divided by the maximum tumor diameter. The invasive degree of the tumor was divided into adenocarcinoma in situ, micro invasive, and invasive adenocarcinoma. In our study, invasive adenocarcinoma was further divided into lepidic-predominant adenocarcinoma and other types of invasive adenocarcinoma. Clinical stage N0 was defined as no hilar or mediastinal lymphadenopathy. Lymphadenopathy was defined as the shortest axis of lymph node ≥ 1 cm. The lung cancer staging was according to the 8th edition of TNM classification issued by International Union Against Cancer (UICC).

### Statistical analysis

Continuous variables were expressed as mean ± SD, and compared by Student’s t test. Categorical variables were compared by Chi-squared test or Fisher exact test. Multivariate analysis by logistic regression was done using variables with statistical significance in univariate analysis. The cutoff value of tumor size was determined by the receiver operating characteristic (ROC) curve. P values less than 0.05 were considered statistically significant. Statistical analysis was performed by the SPSS software (version 19, IBM, USA). This research was permitted by the ethical committee of the first affiliated hospital of Zhejiang University (Hangzhou, China).

## Results

In the study, we included 221 men and 401 women. The median age was 61 years (range from 26 to 85 years), 74 patients benefitted from segmentectomy while 548 patients from lobectomy. Most patients had tumors located at the right upper lobe (202 patients) while 33 patients had tumors located at the right middle lobe, which was the least. Sixty patients presented with lymph node metastasis (28 patients at N1 stage and 32 patients at N2 stage). No lymph node metastasis was found in the pure GGO group. In the GGO-predominant group, 1 patient had N1 lymph node metastasis. The general characteristics of the patients were listed in Table 1.

**Table 1:** The general characteristics of the patients

Variables	Number	Percentage (%)
Gender		
Male	221	35.5
Female	401	64.5
Age		
< 60	273	43.9
≥ 60	349	56.1
Smoking history		
Yes	132	21.2
No	487	78.3
Missing	3	0.5
CEA level (ng/ml)		
≤ 5	533	85.7
> 5	79	12.7
Missing	10	1.6
Tumor Size (cm)		
≤ 1	120	19.3
> 1 and ≤ 2	310	49.8
> 2 and ≤ 3	192	30.9
Lobe distribution		
Right upper lobe	202	32.5
Right middle lobe	33	5.3
Right lower lobe	81	13.0
Left upper Lobe	177	28.5
Left lower lobe	129	20.7
Operation type		
Segmentectomy	74	11.9
Lobectomy	548	88.1
Tumor location		
Central	20	3.2
Peripheral	602	96.8
Consolidation tumor ratio		
Pure GGO	146	23.5
GGO-predominant	163	26.2
Solid-predominant	62	9.9
Solid	251	40.4
Pleural invasion		
Yes	62	10.0
No	560	90.0
Pathologic type		
MIA	34	5.5
LPA	89	14.3
IA	499	80.2
N stage		
N0	562	90.4
N1	28	4.5
N2	32	5.1

*GGO* ground grass opacity; *MIA* micro invasive adenocarcinoma; *LPA* lepidic-predominant adenocarcinoma; *IA* invasive adenocarcinoma

**Fig. 1:**
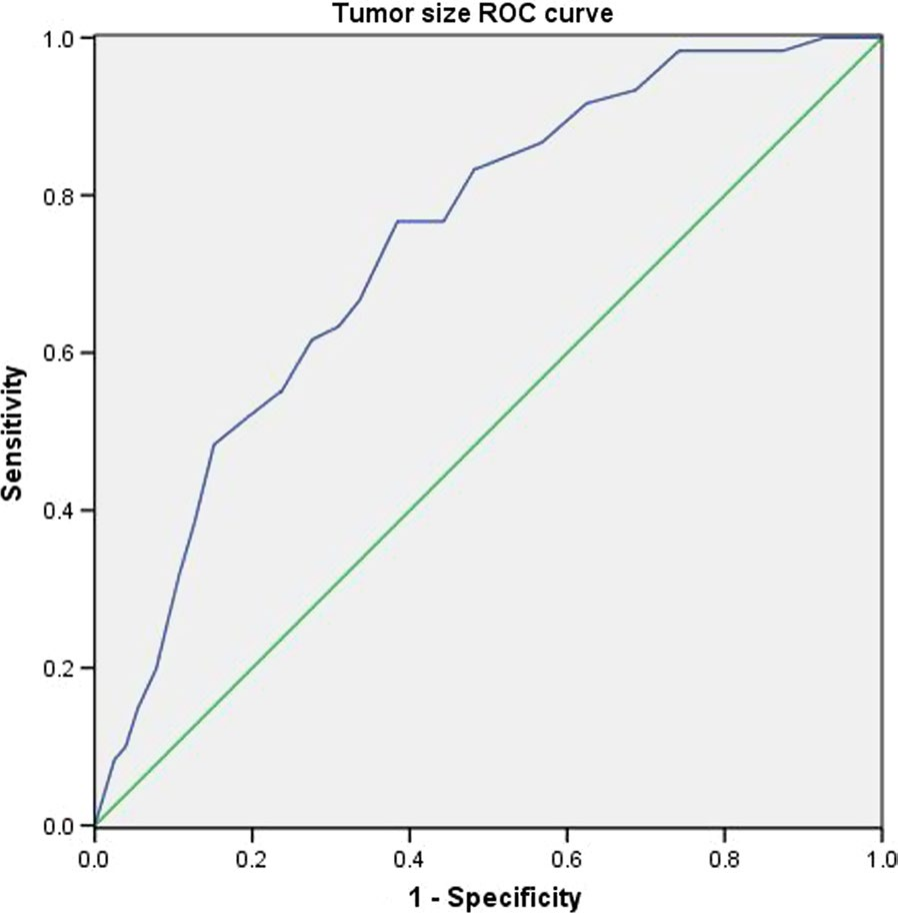
Receiver operating characteristic (ROC) curve of tumor size

Univariate analysis showed that the smoking history, preoperative CEA level, tumor size, tumor location (peripheral or central), consolidation/tumor ratio, pleural invasion and pathologic type were related to lymph node metastasis. Gender, age, and lobe distribution of the tumor did not correlate with lymph node metastasis (Table 2). Preoperative CEA level, tumor size, and consolidation/tumor ratio were found to be independent risk factors in multivariate analysis (Table 3). ROC curve was used to determine the cutoff value of tumor size to predict lymph node metastasis (Fig. 1). The result showed that the cutoff value of tumor size was 1.7 cm and the area under curve (AUC) were 0.74 (*p* < 0.001, 95% CI 0.68–0.80).

**Table 2:** Risk factors of lymph node metastasis by univariate analysis

Variables	N0	N1 + N2	*P* value
Gender			0.296
Male	196	25	
Female	366	35	
Age			0.715
< 60	248	25	
≥ 60	314	35	
Smoking history			0.017
Yes	112	20	
No	447	40	
CEA level (ng/ml)			< 0.001
≤ 5	499	34	
> 5	53	26	
Tumor size (cm)	1.64 ± 0.62	2.17 ± 0.56	< 0.001
Lobe distribution			0.748
Right upper lobe	186	16	
Right middle lobe	30	3	
Right lower lobe	74	7	
Left upper Lobe	159	18	
Left lower lobe	113	16	
Tumor location			0.008
Central	14	6	
Peripheral	548	54	
CTR			< 0.001
≤ 0.5	308	1	
> 0.5	254	59	
Pleural invasion			< 0.001
Yes	46	16	
No	516	44	
Pathologic type			< 0.001
MIA	34	0	
LPA	89	0	
IA	439	60	

*CTR* consolidation/tumor ratio; *MIA* micro invasive adenocarcinoma; *LPA* lepidic-predominant adenocarcinoma; *IA* invasive adenocarcinoma

**Table 3:** Independent risk factors of lymph node metastasis by multivariate analysis

Variables	Odds ratio	95% CI	*p* value
CEA level	4.23	2.21–8.12	< 0.001
Tumor size	2.27	1.37–3.75	0.001
CTR	49.92	6.81–366.27	< 0.001

*CI* confidence interval; *CTR* consolidation/tumor ratio

**Fig. 2:**
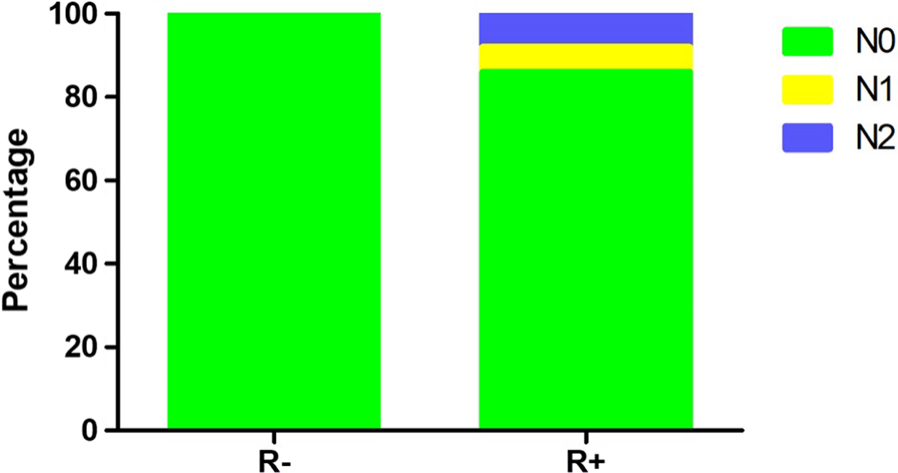
Lymph node metastasis rate of R− and R+ groups

According to the result of multivariate analysis, patients were divided into risk factors positive (R+) and risk factors negative (R−) groups. The R− group was patients with preoperative CEA levels ≤ 5 ng/ml, CTR ≤ 0.5, and tumor size less than 1.7 cm. We included 185 patients in the R− group, and no lymph node metastasis was found. We classified 427 patients into the R+ group and 10 patients were excluded due to lack of CEA level. We found 60 patients with lymph node metastasis in R+ group, 28 patients were staged as N1 while 32 patients were staged as N2. The lymph node metastasis rate was 14% in the R+ group (Fig. 2).

## Discussion

Our research showed that the preoperative CEA level, tumor size, and consolidation/tumor ratio were independent risk factors of lymph node metastasis in lung adenocarcinoma at clinical N0 stage with tumor size ≤ 3 cm, which was in accordance with previous reports. Koike et al. reviewed 894 patients with peripheral clinical stage IA non-small cell lung cancer and reported that the preoperative serum CEA level, tumor size and consolidation/tumor ratio were risk factors. They found patients with preoperative CEA level more than 5 ng/ml had higher rate of lymph node metastasis. The cutoff point of preoperative serum CEA level was 3.5 ng/ml [5]. Simon et al. performed a meta-analysis which included 12 studies with 4666 patients who were clinical stage I involved. They found that higher preoperative CEA level was associated with higher rate of occult lymph node metastasis [8]. Chen et al. retrospectively reviewed 10,885 non-small cell lung cancer patients staged as clinical T1. They divided patients into three groups according to the tumor size (> 0 and ≤ 1 cm; > 1 and ≤ 2 cm; > 2 and ≤ 3 cm). They found that with the increase of tumor size, the rate of lymph node metastasis also increased [19]. In our research, we found that the best cutoff value of tumor size was 1.7 cm. The preoperative CEA level was classified into normal group (≤ 5 ng/ml) or abnormal group (> 5 ng/ml). Consolidation/tumor ratio was also divided into two groups according to the proportion of GGO component (CTR ≤ 0.5 and CTR > 0.5). Preoperative CEA > 5 ng/ml, CTR > 0.5, and tumor size ≥ 1.7 cm were independent risk factors of lymph node metastasis according to the result of multivariate analysis.

The tumor invasive degree was an important pathologic characteristic and is related with the stage of lung cancer. Wang et al. retrospectively analyzed the clinical data of 327 patients with clinical stage IA peripheral lung cancer and the pathologic type was divided as adenocarcinoma in situ (AIS), minimally invasive adenocarcinoma (MIA), and invasive adenocarcinoma (IA). Patients in the AIS and MIA group had no lymph node metastasis, and 26 (10.6%) patients had positive lymph nodes in IA group. Tumor invasive degree was found to be an independent risk factor of lymph node metastasis [20]. In our study, we excluded patients with adenocarcinoma in situ because it was impossible for patients in this group to have lymph node metastasis. Lepidic-predominant adenocarcinoma (LPA) was reported to have lower tumor metastasis and better survival rate than other types of invasive adenocarcinoma, such as acinar predominant adenocarcinoma (APA), papillary predominant adenocarcinoma (PPA), micropapillary predominant adenocarcinoma (MPA) and solid predominant adenocarcinoma (SPA) [21]. We divided patients with invasive adenocarcinoma into LPA group and other types of invasive adenocarcinoma. In our study, patients in the MIA and LPA group had no lymph node metastasis and the lymph node metastasis rate was significantly lower than for other types of invasive adenocarcinomas. However, the tumor invasive degree was not an independent risk factor in multivariate metastasis. This may be because the tumor invasive degree correlated with consolidation/tumor ratio. In pure GGO and GGO-predominant group (CTR ≤ 0.5), 79 (25.6%) patients had LPA and 33 (10.7%) patients had MIA, while in solid predominant and solid group (CTR > 0.5), 10 (3.2%) patients were LPA and only 1 (0.3%) patient with MIA, which was significantly less than the former group (*p* < 0.001). The tumor invasive degree was not found to be an independent risk factor in multivariate analysis when analyzed together with the consolidation/tumor ratio.

Occult lymph node metastasis is not rare in clinical N0 lung cancer [14, 22]. The preoperative radiologic examinations were not reliable in predicting lymph node metastasis according to the research reported by Liberman et al. [4]. However, most patients with clinical N0 stage do have no lymph node metastasis. Wang et al. reported that 7.95% of the clinical stage IA patients had lymph node metastasis [20]. In our study, only 60 of 622 patients had lymph node metastasis and the lymph node metastasis rate was 9.6% in patients with clinical N0 stage. Lymph node dissection was unessential in many patients with clinical early stage for the low lymph node metastasis rate. According to the result of multivariate analysis, we further divided patients into risk factors positive (R+) group and risk factors negative (R−) group. Patients with preoperative CEA level ≤ 5 ng/ml, CTR ≤ 0.5, and tumor size less than 1.7 cm were classified into R− group. There were 185 patients in the R− group and no lymph node metastasis was found. 60 out of 427(14.1%) patients were found to have lymph node metastasis in R+ group. According to the results, we considered that the lymph node metastasis rate was extremely low in clinical N0 lung adenocarcinoma patients with preoperative CEA level ≤ 5 ng/ml, CTR ≤ 0.5 and tumor size less than 1.7 cm. Lymph node dissection was unessential for these patients and should be avoided to reduce surgical trauma because lymph node dissection prolonged operative time, increased blood loss and might lead to postoperative complications such as lymphatic leakage.

This study had some limitations. First, it was a retrospective study and conducted in a single center. Second, PET-CT was performed in only a small number of patients for its high cost. If further multicenter randomized trial including routine PET-CT examination can be conducted, the results will be more convinced.

## Conclusions

Our study demonstrated that preoperative CEA level, tumor size and consolidation/tumor ratio were independent risk factors of lymph node metastasis in clinical N0 lung adenocarcinoma with tumor size ≤ 3 cm. Lymph node metastasis rate was extremely low in clinical N0 lung adenocarcinoma patients without risk factors and lymph node dissection should be avoided in these patients to reduce surgical trauma.

## Data Availability

The clinical data can be achieved in the electronic medical record system in the first affiliated hospital of Zhejiang university.

## Abbreviations

CT: Computer tomography
PET/CT: Positron emission tomography/computed tomography
GGO: Ground grass opacity
CEA: Carcinoembryonic antigen
CTR: Consolidation/tumor ratio
MIA: Micro invasive adenocarcinoma
LPA: Lepidic-predominant adenocarcinoma
IA: Invasive adenocarcinoma
ROC: Receiver operating characteristic
AUC: Area under curve
